# Nuclear localization of histamine receptor 2 in primary human lymphatic endothelial cells

**DOI:** 10.1242/bio.059191

**Published:** 2022-07-01

**Authors:** Sarit Pal, Anatoliy Gashev, Debarshi Roy

**Affiliations:** 1 Department of Medical Physiology, College of Medicine, Texas A&M University, Bryan, TX 77843, USA; 2 Department of Biological Sciences, Alcorn State University, Lorman, MS 39096, USA

**Keywords:** GPCR, Histamine receptor 2, Nuclear localization

## Abstract

Histamine exerts its physiological functions through its four receptor subtypes. In this work, we report the subcellular localization of histamine receptor 2 (H2R), a G protein-coupled receptor (GPCR), which is expressed in a wide variety of cell and tissue types. A growing number of GPCRs have been shown to be localized in the nucleus and contribute toward transcriptional regulation. In this study, for the first time, we demonstrate the nuclear localization of H2R in lymphatic endothelial cells. In the presence of its ligand, we show significant upregulation of H2R nuclear translocation kinetics. Using fluorescently tagged histamine, we explored H2R-histamine binding interaction, which exhibits a critical role in this translocation event. Altogether, our results highlight the previously unrecognized nuclear localization pattern of H2R. At the same time, H2R as a GPCR imparts many unresolved questions, such as the functional relevance of this localization, and whether H2R can contribute directly to transcriptional regulation and can affect lymphatic specific gene expression. H2R blockers are commonly used medications that recently have shown significant side effects. Therefore, it is imperative to understand the precise molecular mechanism of H2R biology. In this aspect, our present data shed new light on the unexplored H2R signaling mechanisms.

This article has an associated First Person interview with the first author of the paper.

## Abstract (precis)

**Summary:** We report the nuclear localization of the histamine receptor 2 (H2R) in lymphatic endothelial cells and other cell types and ligand-dependent effects on H2R nuclear localization kinetics, with histamine-H2R binding serving as a critical step.

## INTRODUCTION

Histamine as a bioactive amine exerts its function through its G protein-coupled receptor (GPCR) subtypes, namely HR1-4 (Thurmond et al., 2008; O'Mahony et al., 2011). Histamine receptor 2 (H2R), widely known for stimulation of gastric acid secretion (Richardson, 1978), is also distributed in various tissue types, such as the blood and lymphatic vascular system (Luo et al., 2013; Fox and Von Der Weid, 2002), immune system (Jutel et al., 2001; Meghnem et al., 2021; Pal et al., 2020a) and nervous system (Vizuete et al., 1997). The bimodal function of histamine is mainly regulated by the activation of H2R and H1R; for example, H1R activation enhances lymphatic pumping whereas H2R induces vessel relaxation (Fox and Von Der Weid, 2002; Kurtz et al., 2014), and H1R enhances Th1-type response whereas H2R activation negatively regulates both Th1- and Th2-type immune responses (Jutel et al., 2001). Furthermore, this differential regulation pattern can also be seen in mast cell (MC) degranulation (Pal et al., 2020b; Carlos et al., 2006; Pal, 2019). Overall, mechanistic understanding of H2R's downstream signaling, its subcellular localization as a GPCR and, specifically, its significance in lymphatic physiology remain elusive.

Generally, GPCRs are presumed to be localized in the plasma membrane, and their signaling cascades mediate through the engagement of the G proteins. However, an emerging number of studies have shown that GPCR localization can be observed in subcellular organelles, such as in the endoplasmic reticulum (Petäjä-Repo et al., 2000; Lumaret et al., 2007), Golgi apparatus (Yudowski et al., 2009) and nucleus. For example, nuclear-localized GPCRs include prostaglandin E2 receptor (Bhattacharya et al., 1998), oxytocin receptors (Kinsey et al., 2007; Di Benedetto et al., 2014), CysLT1 receptor (Nielsen et al., 2005), sphingosine 1 phosphate receptor subtype 1 (Estrada et al., 2009), chemokine receptor CCR2 (Favre, 2008) and beta 1 adrenergic receptor (Vaniotis et al., 2011; Boivin et al., 2006).

In this study, we demonstrate the unique nuclear localization pattern of H2R in human lymphatic endothelial cells (LECs). To show that H2R localization is not limited to LECs, we have studied H2R localization in other primary cell lines, human intrahepatic biliary epithelial cell (HIBECs), lymphatic fibroblasts, rat cholangiocytes and rat mesenteric perilymphatic tissues. We explored the colocalization of H2R with its signaling partner G alpha S (Wellner-Kienitz et al., 2003), as well as with nuclear envelope marker Lamin A. We demonstrate the nuclear translocation kinetics of H2R in response to its ligand and the effect of this translocation during pharmacological blockade of H2R. Furthermore, using fluorescently tagged histamine, our data suggest that the binding of histamine with H2R positively regulates the translocation event. Finally, we performed basic local alignment search tool (BLAST) analysis, and found that beta 1 adrenergic receptor, which is reported to be localized in the nucleus, shares significant sequence identity with H2R.

Altogether, our findings reveal not only the previously unrecognized localization pattern of H2R but also point out critical questions regarding the functional importance of H2R nuclear localization as a member of GPCR families. Furthermore, whether H2R nuclear localization has discrete transcriptional regulation in the context of certain pathophysiology remains elusive. For example, contribution towards lymphatic contractile dysfunction during chronic inflammation or expression of certain adhesion molecules in LECs affecting immune cell trafficking has yet to be explored. Finally, H2R blockers are one of the most widely used over-the-counter drugs; however, recent evidence demonstrates the side effects of their use. For example, ranitidine, a commonly used H2R blocker used for gastrointestinal ulcers, has recently been shown to be associated with gastrointestinal cancer (Sabesin, 1993; Shi et al., 2019; Mcgwin, 2020). These evidence together strongly suggest the present knowledge gap in the mechanistic understanding of the histamine-H2R signaling axis. Our present finding of unique H2R localization pattern will enhance the current scope of H2R biology in this context.

## RESULTS

### H2R nuclear localization in LECs and in different cell and tissue types

LECs are present in the inner linings of the lymphatic vessel lumen and significantly contribute toward immune cell trafficking during inflammatory responses (Pal et al., 2020a,b). Recently, we along with others have demonstrated the responsiveness of lymphatic vessel contractile function, such as contraction frequency, and pumping efficiency towards histamine or its receptor antagonists (Fox and Von Der Weid, 2002; Kurtz et al., 2014). However, the expression of H2R, specifically in lymphatic endothelial cells remains unclear.

**Fig. 1.**
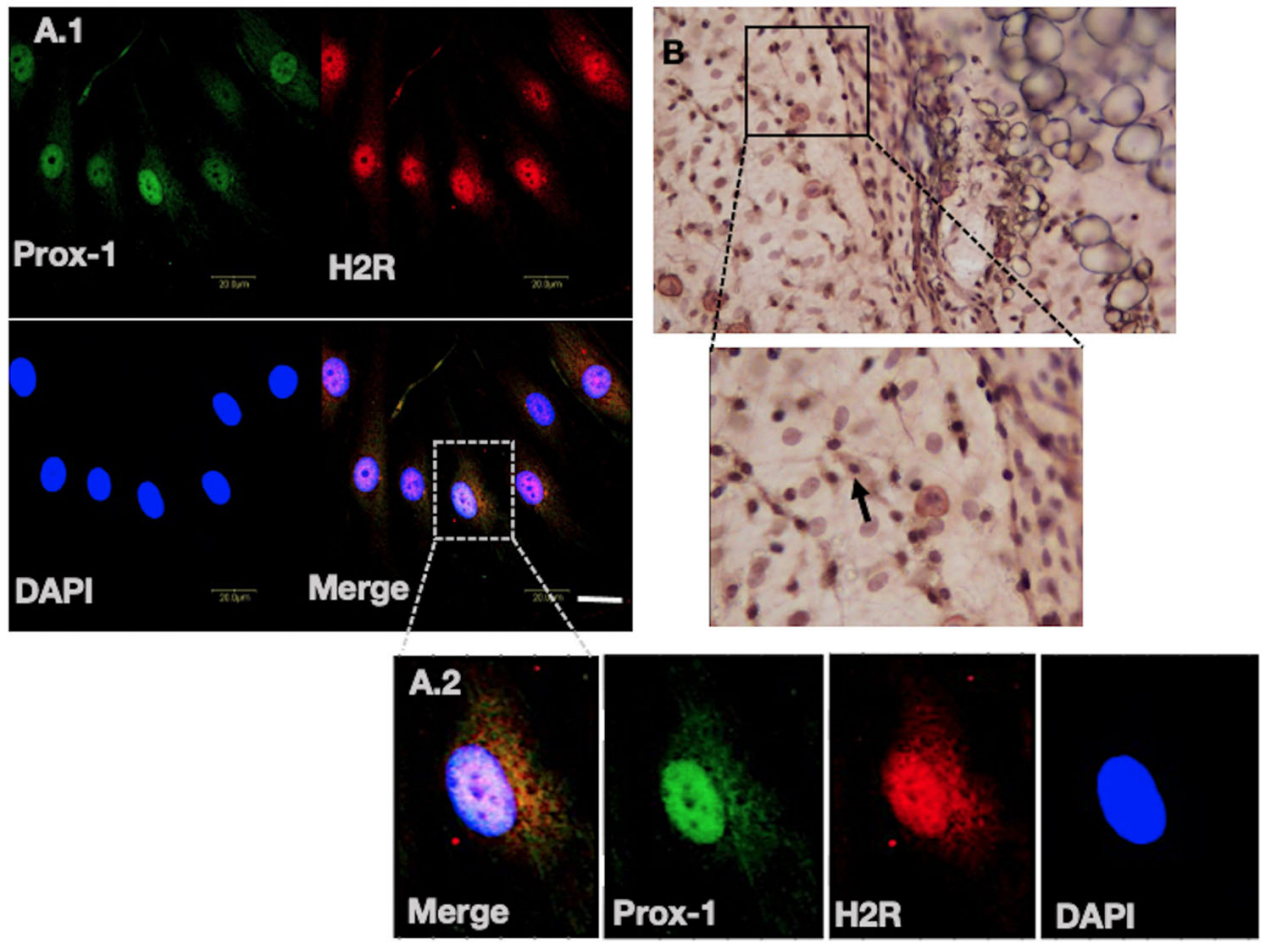
**Nuclear localization of H2R.** (A.1) Confocal images of LECs, labeled for Prox-1 (green channel), H2R (red channel), nuclei (DAPI) and overlay of all three channels. (A.2) Inset of one cell zoomed, which indicates the localization of H2R, Prox-1 and DAPI. (B) IHC staining performed on the rat perilymphatic tissue. The tissue was stained with H2R antibody, showing its nuclear localization; the inset zoomed view shows the localization pattern. Images are representative of at least three independent experiments. Scale bar: 20 µm.

In this study, our data on primary LECs demonstrate the expression of H2R (Fig. 1A.1) and its distinct localization pattern in the nucleus (Fig. 1A.1). To investigate further, LECs were co-immunostained with lymphatic specific marker Prox-1, a transcription factor, as well as with 4′,6-diamidino-2-phenylindole (DAPI), a nuclear marker. The co-immunofluorescence data show the localization of H2R with DAPI and Prox-1 in the nucleus (Fig. 1A.1,A.2). Further to verify this H2R localization pattern, we studied other primary cell types, such as human primary lymphatic fibroblasts (Fig. S2A.1), HIBECs (Fig. S1B.1) and primary rat cholangiocytes (Fig. S2C.1). For HIBECs and rat cholangiocytes, we used a specific marker, CK19, and for lymphatic fibroblasts Prox-1. Negative control, absence of H2R primary antibody during immunofluorescence (IF) staining, is provided in LECs (Fig. S1A.1-4) and in HIBECs (Fig. S1C.1-4). Overall, LECs, along with HIBECs, rat cholangiocytes and human lymphatic fibroblasts, showed discrete nuclear localization of H2R. Based on this observation, we also explored H2R localization in whole tissue segments using immunohistochemistry (IHC) in rat mesenteric perilymphatic tissue (Fig. 1B), and IF staining of H2R in perilymphatic MCs (MCs stained with Avidin) (Fig S2C.1-3) and rat liver sections (Fig S2D.1-2). The positive H2R staining in the nucleus in rat tissues corroborates with the H2R nuclear localization pattern in cultured primary human LECs, as well as other primary cell lines, reinforcing the present findings.

### Localization of H2R signaling partner G alpha S and BLAST analysis of H2R with nuclear-localized GPCRs

**Fig. 2.**
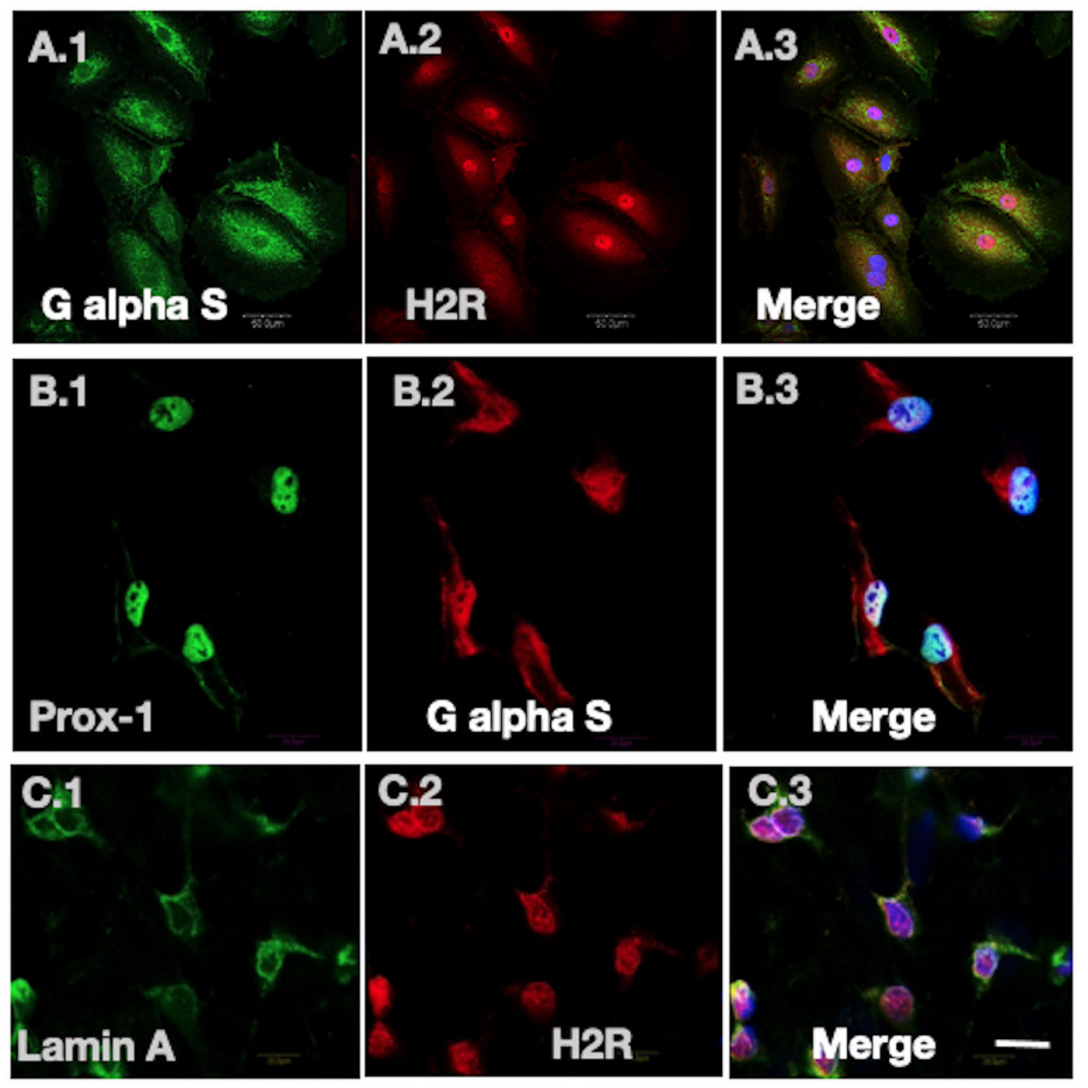
**Colocalization of H2R with signaling partner G alpha S and nuclear envelope marker Lamin A in the nucleus.** (A.1-3) Confocal images of LECs, labeled for G alpha S (green channel, A.1), H2R (red channel, A.2) and overlay merge of G alpha S, H2R and nuclear marker (DAPI) (A.3), showing the colocalization of H2R with signaling partner G alpha S in the nucleus. (B.1-3) LECs labeled with lymphatic specific marker Prox-1 (B.1), G alpha S (B.2) and overlay (B.3), showing expression of G alpha S in LECs. (C.1-3) Confocal images of nuclear envelope marker Lamin A (C.1) and H2R (C.2), demonstrating H2R localization in the nucleus in the overlay image (C.3). Scale bar: 20 µm.

H2R as a GPCR partners with the G protein G alpha S (Monczor and Fernandez, 2016) for its downstream signaling cascade. Therefore, to assess whether G alpha S also localizes with H2R in the nucleus, IF staining of Prox-1 with G alpha S and G alpha S with H2R were done in LECs. Fig. 2B.1-3 demonstrate the expression of G alpha S with Prox-1, an LEC-specific nuclear marker. Subsequent immunostaining data of H2R (in red channel) and G alpha S (in green channel) show colocalization of G alpha S and H2R (Fig. 2A.1-3) in the nucleus (stained with DAPI). This finding is in agreement with the previous finding of G protein localization in the nuclear membrane and in the intra-nuclear structures in adult cardiomyocytes (Boivin et al., 2006). In addition, we co-immunostained H2R and the nuclear envelope marker Lamin A (Fig. 2C.1-3) in LECs exhibiting distinct H2R localization in the nucleus.

Based on these findings, we further investigated the sequence similarity of H2R with previously demonstrated nuclear localized GPCRs, such as oxytocin receptor, prostaglandin receptor, chemokine receptor, thyroid receptor alpha, estrogen receptor alpha and beta 1 adrenergic receptor, using BLAST tool. Strikingly, we found H2R to have significant sequence similarity (49%) and identicality (34%), with an e value of 3e^−59^ (Figs S3, S4) with beta 1 adrenergic receptor, which has shown to be nuclear localized. In addition, in phylogenetic tree analysis, we found, based on the brunch point distance, that H2R and beta 1 adrenergic receptor share distance similarity between H2R with its closest family member H1R. Altogether, the evolutionarily close relationship, sequence similarity and similar G protein signaling molecule between H2R and beta 1 adrenergic receptor reinforce the present observations of H2R's nuclear colocalization pattern.

### Effect of histamine treatment on H2R nuclear translocation

**Fig. 3.**
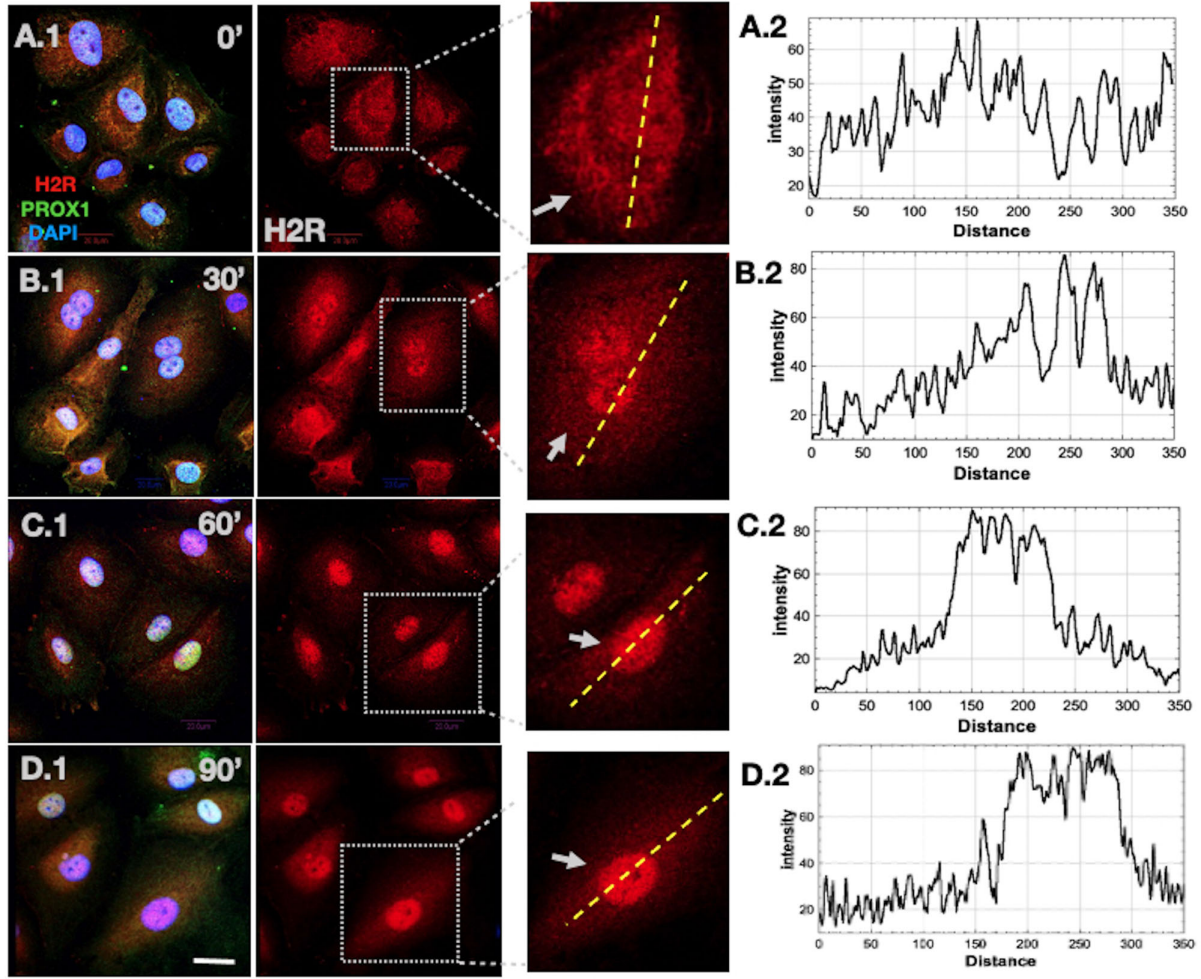
**Ligand-induced H2R nuclear translocation kinetics in LECs.** (A.1-D.2) LECs were incubated with histamine at different timepoints, and nuclear localization of H2R was analyzed by line scan graphs. A.1 (0′), B.1 (30′), C.1 (60′) and D.1 (90′) show overlay of Prox-1, H2R and DAPI; middle panels show staining with H2R; A.2 (0′), B.2 (30′), C.2 (60′) and D.2 (90′) show the corresponding line scan of a cell from the inset, which indicates relative fluorescent intensity (arbitrary unit) in the cytoplasm and nucleus. Arrows indicate the pattern of H2R localization. All the images are representative of at least three independent experiments. Scale bar: 20 µm.

**Fig. 4.**
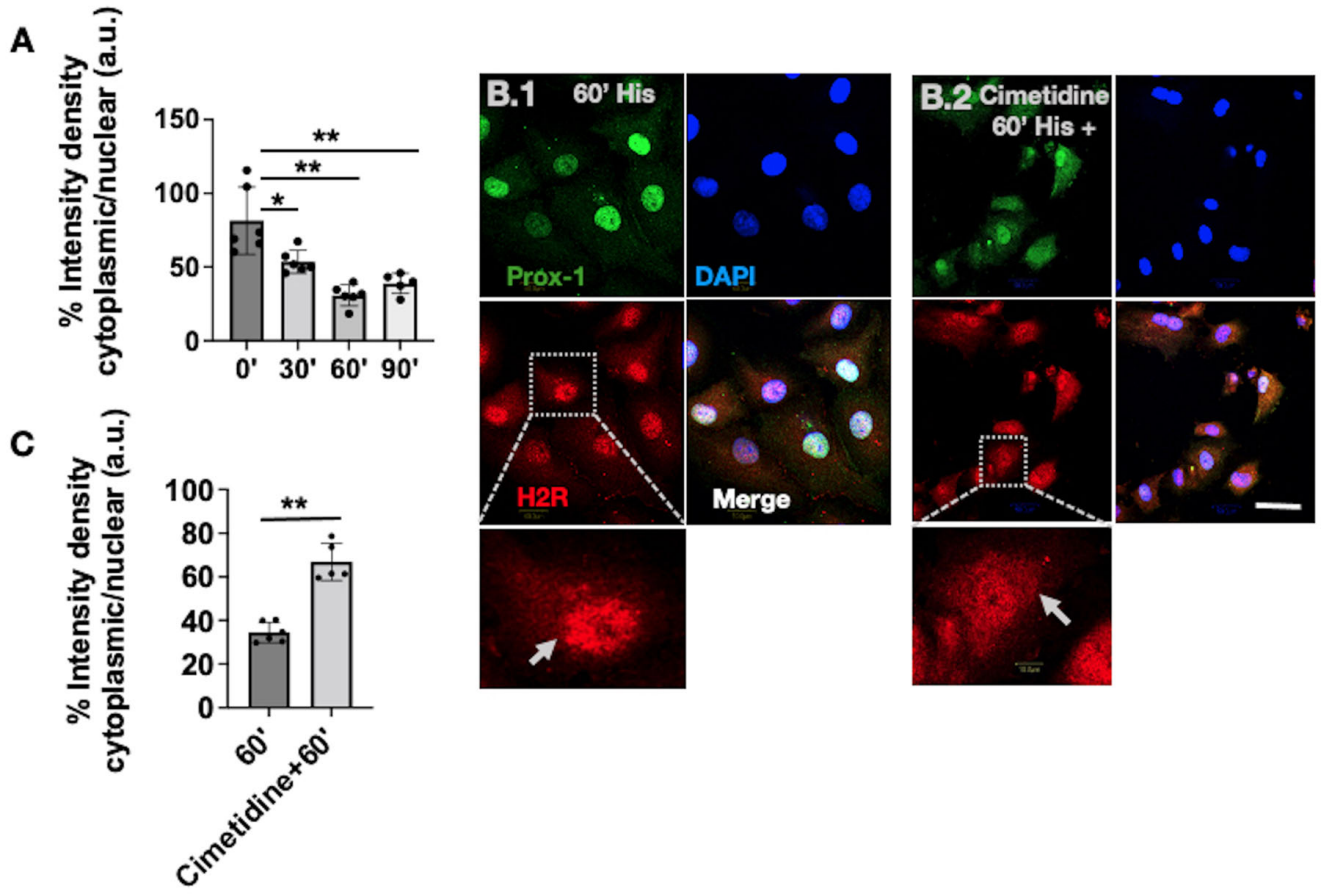
**Effect of H2R blockade on H2R nuclear translocation.** (A) Bar graph represents the percentage quantification of histamine-induced cytoplasmic versus nuclear H2R intensity density between 0′, 30′, 60′ and 90′ groups. (B.1) Confocal images of LECs, treated with histamine for 60′; H2R staining is in the red channel, with the inset showing translocation of H2R into the nucleus. (B.2) LECs treated with cimetidine and histamine. Inset shows translocation of H2R after treatment. (C) Quantification of the treatment of 60′ histamine and cimetidine+60′ histamine group. Arrows indicate the pattern of H2R localization. All the images are representative of at least three independent experiments. Scale bar: 20 µm. *P<0.01, **P<0.001. a.u., arbitrary units.

To elucidate whether histamine treatment can induce H2R's nuclear localization and can alter the ratio between perinuclear versus nuclear H2R intensity density, as previously shown in nuclear-localized GPCRs (Nielsen et al., 2005), LECs were treated with 10 µM histamine at 0 min (′) (without histamine stimulation), 30′, 60′ and 90′ timepoints. The histamine concentration was chosen based on our previous study and a study by Kurtz et al. (Kurtz et al. 2014; Pal et al. 2020a). Within 30′ after histamine treatment, we observed increased nuclear translocation of H2R compared to 0′ control. Subsequently, 60′ and 90′ histamine treatment resulted in a significant increase in nuclear H2R intensity density compared to 30′ and 0′ control as well (Fig. 3A.1-D.1). This H2R translocation kinetics was quantified by ImageJ by measuring the cross-sectional intensity plot of a cell as previously demonstrated by Kocanova et al. (Kocanova et al., 2010). Precisely, a line scan cross-section was selected, which comprises the entire cytoplasm and nucleus. The line scan intensity histogram (Fig. 3C.2) of 60′ and 90′ (Fig. 3D.2) groups demonstrated a significant increase and focused H2R fluorescence around the nuclear region compared to the perinuclear region. However, in the 0′ control group, the intensity histogram of nuclear region (Fig. 3A.2) is minimally above the cytoplasmic region, suggesting that the 0′ control group has higher cytoplasmic H2R intensity compared to the groups treated with histamine for 30′, 60′ or 90′. The quantification of cytoplasmic versus nuclear H2R fluorescence intensity of 0′ control, 30′, 60′ and 90′ is shown in Fig. 4A. The data suggest that the untreated control or 0′ timepoint has the highest H2R cytoplasmic/nuclear intensity density, and increasing histamine treatment timepoints such as 30′, 60′ and 90′ show significantly diminished cytoplasmic/nuclear intensity, suggesting histamine-dependent effect of H2R nuclear translocation.

Based on these results from Fig. 3A.1-D.2 and Fig. 4A, we further investigated the effect of pharmacological blockade in H2R nuclear translocation event. In the 60′ His+cimetidine group, LECs were pretreated with 100 µM cimetidine (Kurtz et al., 2014) for 60′ and subsequently with histamine and cimetidine for 60′. For the control, we treated LECs with histamine for 60′ (Pal et al., 2020a). The 60′ timepoint was chosen as this timepoint showed the highest H2R nuclear translocation compared to 30′ and 90′. Fig. 4B.1 suggests that histamine-dependent H2R activation induces H2R nuclear translocation in 60′; however, H2R blockade by cimetidine, shown in Fig. 4B.2, inhibited H2R nuclear translocation. Fig. 4C shows the quantification of H2R cytoplasmic/nuclear fluorescence intensity density, where cimetidine treatment inhibited H2R translocation, causing an increase in H2R cytoplasmic fraction and thereby a significant increase in H2R cytoplasmic/nuclear intensity density compared to the 60′ treatment group. Overall, these data suggest that ligand-dependent activation of H2R plays a critical role in H2R nuclear translocation kinetics.

### H2R binding with its ligand is crucial for its nuclear translocation

**Fig. 5.**
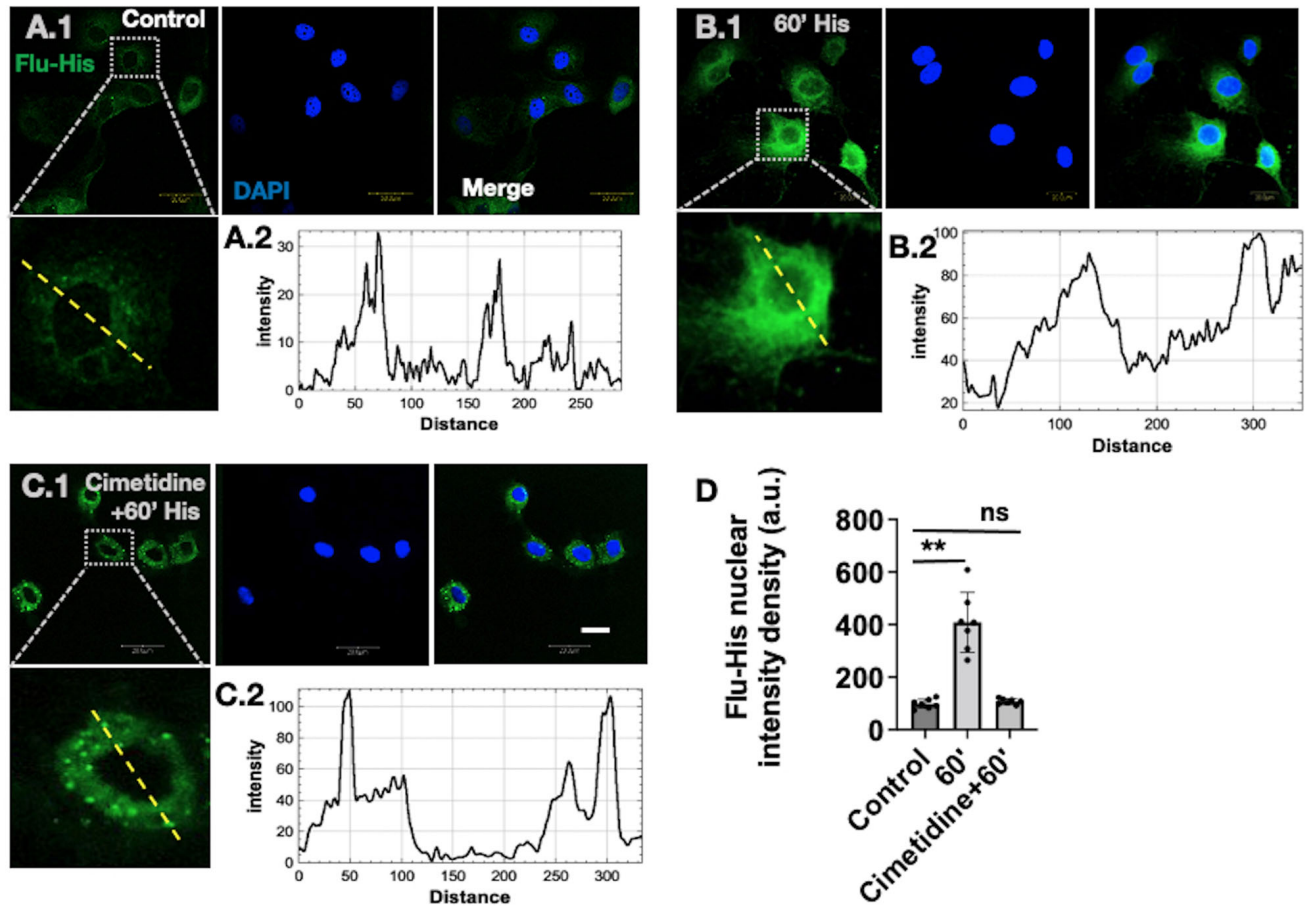
**Inhibition of H2R by cimetidine treatment attenuates H2R nuclear translocation.** (A.1) Confocal images of LEC 0′ control group. Inset shows the line scan on the respective cell. (A.2) Based on that, the respective line scan intensity graph was generated. (B.1) LECs treated with Flu-His molecule for 60′. (B.2) The intensity graph of a line scan from the respective cell shown in the inset. (C.1) Images of LECs pretreated with cimetidine for 60′ and subsequently treated with Flu-His for 60′. (C.2) The intensity graph of the line scan from the respective cell shown in the inset, showing Flu-His signal intensity. (D) Quantification of nuclear intensity density of 0′ control, 60′ Flu-His and cimetidine+60′ Flu-His groups. All the images are representative of at least three independent experiments. Scale bar: 20 µm. ns, not significant; **P<0.001.

To further investigate whether histamine-H2R binding interaction plays a role in nuclear localization, we treated LECs with fluorescently tagged histamine (Flu-His) probe as shown in previous GPCR localization studies (Nielsen et al., 2005). Three treatment groups were chosen: control group, LECs were briefly exposed to fluorescently tagged histamine solution; 60′ histamine group, LECs were incubated with Flu-His for 1 h; and cimetidine+60′ His group, LECs pretreated with cimetidine for 1 h followed by 1 h of Flu-His tracer treatment. We investigated in the 60′ timeframe as histamine-induced H2R nuclear localization was highest in 60′ compared to other timepoints. We used the cimetidine+60′ His group to elucidate whether blockade of H2R affects Flu-His-H2R binding and subsequent nuclear localization. The results in Fig. 5 show the control group has significantly diminished fluorescent intensity, quantified in the line scan graph (Fig. 5A.1,A2), compared to the 60′ and cimetidine+60′ groups (Fig. 5B.1-2,C1-2). The 60′ Flu-His treatment group exhibited significant localization of florescent signal into the nucleus; however, in the cimetidine treatment group, the signal intensity of fluorescently tagged histamine in the nuclear region was significantly reduced. The line scan histogram in Fig. 5B.2 for the 60′ group has a comparatively higher Flu-His intensity density in the nuclear region compared to the cimetidine+60′ group. However, in the cimetidine+60′ group, aggregations or puncta of Flu-His molecules were observed in the cytoplasmic region (Fig. 5A.1-C.2), which were not observed in either the control or 60′ group. The quantification of nuclear intensity density is presented in Fig. 5D. The bar graph shows that the 60′ group exhibits 4-fold higher nuclear intensity density compared to the 0′ control and cimetidine+60′ groups. This recapitulates the similar trends of line histogram plots, suggesting that H2R-Histamine binding is critical for subsequent nuclear translocation of H2R.

## DISCUSSION

H2R is expressed in multiple organ systems and its activation corresponds to several pathophysiological states (O'Mahony et al., 2011). However, H2R's precise signaling mechanism and structure-function relationship between the H2R-histamine complex remain unknown. In the context of lymphatic circulation, previous studies have shown the significance of H2R in contractile responses of collecting lymphatic vessels; however, its expression specifically in LECs has remained elusive (Fox and Von Der Weid, 2002; Kurtz et al., 2014). Our present data show the expression of H2R in LECs but, in addition, demonstrate the unique localization pattern of H2R in the nucleus. We identified that this localization is not only restricted to LECs but is also observed in other primary human cell lines and mesenteric perilymphatic and liver tissue segments. We show nuclear colocalization of H2R along with its signaling partner G alpha S and nuclear envelope marker Lamin A. Similar to other nuclear-localized receptors, we further identified that a 1-h histamine treatment can significantly induce H2R nuclear translocation and, moreover, H2R pharmacological blockade can attenuate this nuclear shuttling. On the basis of these findings, using fluorescently tagged histamine, we further demonstrated that H2R-Histamine binding interaction is crucial for H2R's nuclear localization. Finally, with protein BLAST, we identified that H2R and beta 1 adrenergic receptor, which also localizes in the nucleus, share 34% sequence identity, 49% sequence similarity and have 77% query coverage, with a significant e value of 3e^−59^. Altogether, the present experimental evidence along with the bioinformatic analysis reinforce the present finding of H2R's nuclear localization. On the other hand, several pertinent questions were unable to be addressed in this work. For example, the nuclear translocation of any protein is dependent upon its nuclear localization sequence (NLS). As the H2R crystal structure is not resolved yet, the NLS was particularly difficult to determine. Using fluorescently tagged H2R construct with or without NLS in the presence of histamine could perhaps complement the present IF data.

The present observations not only provide evidence on the increasing list of nuclear-localized GPCRs but also prompt several unexplored questions. For example, in receptor biology, when a ligand shares multiple cognate receptors and when those receptors are simultaneously expressed in a certain cell type, how is the binding of that ligand to one receptor over another determined? Is this binding interaction stochastic or gradient dependent in nature? Furthermore, how does the intrinsic state of the cell or extrinsic microenvironment shape the preferential expression of one subtype of the receptor over others (Pope and Medzhitov, 2018; Ben-Shlomo and Hsueh, 2005)? For example, among four HRs, MCs and Th cells express both H1R and H2R regulating specific downstream signaling networks. As both the receptors are simultaneously expressed, how is the binding of histamine preferred for H1R over H2R or vice versa? How is the expression of those receptors being regulated? Several similar unresolved questions underscore the need for a deeper understanding of subcellular localization and the mechanism of gene expression patterns where the same ligand activates multiple receptors. Our finding of H2R's nuclear localization provides a possibility that histamine, as a widely synthesized molecule in several cell and tissue types, can differentially regulate the expression and signaling networks based on their receptor subcellular localization. In particular, the localization of H2R in the nucleus, like other nuclear-localized GPCRs (Boivin et al., 2006), can be beneficial for the responsiveness towards the transcriptional regulation of a specific set of genes based on the external cue or homeostatic or pathological state of the cell. This study also brings a further set of unexplored questions, such as what is the functional and physiological relevance of H2R nuclear localization in the context of lymphatic physiology? If H2R is capable of transcriptional regulation, what are the downstream genes associated with H2R transcriptional regulation directly or indirectly affecting lymphatic function? As binding with histamine with H2R induces H2R nuclear localization, is histamine-H2R binding alone sufficient for this translocation or is there a contribution of chaperones in this event? As a whole, our unique findings underscore the unexplored biology of H2R as a nuclear-localized GPCR and open up previously unrecognized signaling networks in lymphatic vasculature as well in broader pathophysiological contexts.

## MATERIALS AND METHODS

### Cell culture and materials

Primary LECs (cat #C12217) were procured from Promocell, Heidelberg, Germany, and cultured with a MV2 media kit (cat #C-22022, Promocell). HIBECs (cat #5100) were procured from Sciencell, Carlsbad, CA, USA, and cultured with Epi CM (cat #4101, Sciencell). Lymphatic fibroblasts (cat #2530) were procured from Sciencell and cultured with medium (cat #2301, Sciencell). Rat primary cholangiocytes were grown in Nunc 35-mm glass-bottom dishes (SKU #801001, Nest Scientific USA, Woodbridge, NJ, USA) with appropriate media and cultured as described previously (Flister et al., 2010). When cells were confluent, Trypsin-EDTA (0.05%) solution was used (cat #25300054, Life Technologies) for cell splitting. The following chemical reagents were used for the experiments: histamine dihydrochloride (cat #H7250, Millipore Sigma), HR2 antagonist, cimetidine (cat #C4522, Millipore Sigma), Triton X-100 (cat #T8787, Sigma Aldrich), fluorescently tagged histamine, EverFluor FL histamine (cat #7148, Setarah Biotech, Eugene, OR, USA), phosphate-buffered saline (PBS; 10, cat #6505, EMD Chemicals, Gibbstown, NJ, USA)

### IF staining

All the primary cells used for experiments were between passage number 2 and 4. Cells were grown in Nunc glass-bottom dishes (cat #150680, Thermo Fisher Scientific). After reaching 80-90% confluence, cells were fixed in paraformaldehyde (1% w/v in PBS, pH 7.4) for 15 min, washed three times in PBS and incubated with blocking solution (5% goat serum, 0.1% Triton X-100 in PBS) for 60 min at room temperature. For LECs and lymphatic fibroblasts, Prox-1 was used as a lineage marker, and for HIBECs and rat cholangiocytes, CK-19 was used as a lineage marker. Cells were incubated with primary antibodies (with a dilution of 1:100) overnight at 4°C, followed by washing three times, subsequent secondary antibody (1:200 dilution) staining for 1 h, washing three times, and mounting with Prolong glass antifade mounting reagent conjugated with DAPI for nuclear staining. For the negative control, all the steps were followed except the addition of H2R primary antibody during primary antibody staining. Images were acquired using the same acquisition settings for all slides (here and below) on an Olympus Fluoview 300 confocal microscope with a 40× water objective with 1.15 numerical aperture, and step size of 0.5 μm in 488, 647 and 405 nm laser lines. For IF studies, the following H2R primary antibodies were used: cat #NB600812 (Novus Biologicals; 1:150 dilution) (Pal et al., 2020a) and cat #AHR 002 (Almone Laboratories, Jerusalem, Israel; 1:200 dilution) (Pal et al., 2020a). For rat peri lymphatic and liver segment IHC, the following H2R antibodies were used: cat #NLS1175, (Novus Biologicals; 1:50) and cat #ab188933 (Abcam; 1:50). Other antibodies used were as follows: G alpha S (cat #ab 235956, Abcam; 1:200), Lamin A (cat # MA1-5820, Invitrogen; 1:200) and Prox-1 (cat #ab199359, Abcam; 1:200). Alexa fluor 647 goat anti-rabbit HL (cat #A-21245), Alexa fluor 488 goat anti-mouse IgG1 (cat #A-21121), Alexa fluor 647 goat anti-mouse HL (cat #A-21236) (all 1:500 dilution) and ProLong Glass Antifade Mountant with NucBlue Stain (cat #P36985) were from Thermo Fisher Scientific. Blocking goat serum (cat #005-000-121) was procured from Jackson ImmunoResearch. IF staining in different cell lines and tissues was repeated in at least three independent experiments with two replicates each time.

### Surgical isolation of whole mesenteric perilymphatic tissue, IHC and IF staining for whole tissue

Surgical procedure and perilymphatic segment isolation were performed as previously (Pal et al., 2020a). All animal procedures for the current studies were reviewed and approved by the Texas A&M Institutional Animal Care and Use Committee. For the current study, male Sprague-Dawley rats with an average body weight of 200-250 g were used. Briefly, rats were euthanized by an overdose of isoflurane followed by thoracotomy. Immediately the sternum and half of the adjacent ribs were excised, and the inferior vena cava was cut to drain blood. The abdominal cavity was opened by midline abdominal incision, the two ends of the gut included in the area to be excised were sutured before excision to avoid fecal contamination of the preparation, and the root of the mesentery was clamped to minimize further bleeding. The excised gut with attached mesentery was rinsed three times in 1× PBS. For perilymphatic tissue dissection, the gut was pinned down in a Sylgard^®^-coated 10-mm Petri dish submerged in cold physiological salt solution with the pH adjusted to 7.36. The whole mesentery from an individual animal was rapidly excised and, using a dissecting microscope, was separated into segments that included mesenteric lymphatic vessels and perilymphatic tissues, but did not include large segmental mesenteric arteries or veins. Special care was taken to ensure minimal perturbation during tissue dissection (Pal et al., 2020a; Gasheva et al., 2019; Pal, 2019). For IF and IHC staining from one animal, six perilymphatic segments were isolated and processed for each staining methods. Tissues from seven animals were used for IF and IHC staining. The detailed protocol for whole perilymphatic tissue immunofluorescent staining can be found in Pal et al. (2020a). For IHC, perilymphatic tissue segments were incubated overnight at 4°C with H2R antibody (1:50) followed by PBS wash. Subsequently, segments were incubated with a secondary biotinylated antibody at room temperature for 20 min (Dako Cytomation LSAB Plus System-HRP, Glostrup, Denmark). Further incubation was done with Dako ABC for 20 min and developed with 3 diaminobenzidine (Dako Cytomation Liquid DAB Plus Substrate Chromogen System). Tissue segments were imaged with a BX-51 light microscope (Olympus, Tokyo, Japan) with a Videocam (Spot Insight; Diagnostic Instrument, Sterling Heights, MI, USA) and processed with an image analysis system as previously described (Glaser et al., 2014).

### BLAST analysis

Using BLAST, amino acid sequences of H2R were compared with nuclear localized GPCRs. FASTA sequences were used. The accession numbers are as follows: H2R (accession number BAA84279.1), estrogen receptor alpha (ERα) (accession number AAH63795), thyroid hormone receptor alpha (TRα) (accession number BAH02277) (Tsai and O'malley, 1994), prostaglandin e2 receptor (accession number AAA36438), beta 1 adrenergic receptor (accession number ABY87521.1) (Boivin et al., 2006) and oxytocin receptor (accession number NP001341583). For control comparison, BLAST was done between H2R with its isoform 1 (accession number NP_001124527). Multiple sequence alignment values such as query coverage, e value, percent identicality and percent similarity were measured (Ye et al., 2006). Based on the significant e value and sequency identity, phylogenetic tree analysis was done for H2R, beta 1 adrenergic receptor and oxytocin receptor. For control, H1R was incorporated in this analysis.

### Nuclear translocation kinetics of H2R in the presence of histamine

LECs were grown in Nunc 35-mm glass-bottom dishes with appropriate media and cultured up to 80-90% confluence. Cells were washed with PBS and thereafter treated with a final concentration of 10 µM histamine in serum-free media for 30, 60 and 90 min along with untreated control. For histamine receptor 2 blockade, cells were washed and treated with 100 µM cimetidine for 1 h as we used previously (Pal et al., 2020b; Pal, 2019) and subsequently treated with histamine and 100 µM cimetidine for 60 min. After treatment, cells were fixed, washed, immunostained for H2R and Prox-1 with appropriate secondary antibodies and mounted with DAPI-conjugated mounting solution. DAPI staining allowed the detection of nuclear periphery quantification of cytoplasmic and nuclear intensity density. Image acquisition was done using a 40× water immersion objective on an Olympus Fluoview 300 confocal microscope with 1.15 numerical aperture and step size of 0.5 μm under appropriate excitation/emission wavelengths. Further intensity density percentage was calculated using ImageJ. First, the area of the nucleus was identified in DAPI channel, then further intensity density of the area of the nucleus as well as the mean fluorescence intensity (MFI) of the area of the cytoplasm excluding the nucleus was determined in 647-nm channel. Percentage of intensity density was calculated using the formula (cytoplasmic H2R intensity density/nuclear H2R intensity density)*100 for at least four cells from each image; at least two images from three independent experiments were analyzed for statistical significance. The line scan graph was generated as described previously. Briefly, with ImageJ, a cross-sectional line was drawn, which encompasses the cytoplasmic and nuclear region clearly, not overlapping with other cells. Further, using ImageJ analysis window, a line scan cross-sectional graph was generated (Kocanova et al., 2010).

### Fluorescently labeled histamine binding assay

LECs were grown in Nunc 35-mm glass-bottom dishes with appropriate media (MV2 media kit, Promocell) and cultured up to 80-90% confluence in three groups: control, 60′, cimetidine+60′. For the cimetidine+60′ histamine group, LECs were first pretreated with 100 µM cimetidine in serum-free medium, incubated for 1 h, then fluorescently tagged histamine solution (EverFluor FL histamine) was added for a final concentration of 10 µM (Pal et al., 2020a) and incubated for another hour. The 60′ group was treated with a final concentration of 10 µM fluorescently tagged histamine solution and incubated for 1 h. After incubation, both groups were washed with PBS three times for the removal of unbound dyes, subsequently mounted with DAPI-conjugated ProLong Glass Antifade Mountant (cat #P36985), with 1.5 thickness cover glass and immediately proceeded for image acquisition. For the control group, fluorescently tagged histamine solution was briefly added, followed by rinsing with PBS three times, and subsequent steps were followed as mentioned above. Image acquisition was done using a 40× water immersion objective on an Olympus Fluoview 300 confocal microscope with 1.15 numerical aperture and step size of 0.5 μm under appropriate excitation/emission wavelengths. Images were taken from random two to three fields of view from each group. For fluorescence intensity density measurements, the signals were quantified with the National Institutes of Health (NIH) ImageJ program. First, images were converted to an 8-bit grayscale, regions of interest (ROIs) with DAPI were marked on the 408-nm channel, and the MFI of the 488-nm channel was measured on the marked ROIs. Fluorescent signals from at least four cells in each image and at least two images from independent three experiments were analyzed for statistical significance. The image analysis was done as described previously (Pal et al., 2020b; Pal, 2019).

### Statistical analysis

Statistical analysis was performed using a Student's *t*-test or one-way analysis of variance with multiple comparisons to obtain a *P*-value using GraphPad Prism Version 9.3.1. *P*-values are indicated by asterisks (*P<0.01, **P<0.001). All experiments were performed a minimum of three independent times with at least four replicates per group.

## Supplementary Material

Supplementary information
