# Supplementary material for: Nuclear localization of histamine receptor 2 in primary human lymphatic endothelial cells
Source: Biol Open. 2022 Jul 1;11(7):bio059191. doi: 10.1242/bio.059191 (PMC9257380; doi:10.1242/bio.059191)
Supplement: Supplementary information [file biolopen-11-059191-s1.pdf]

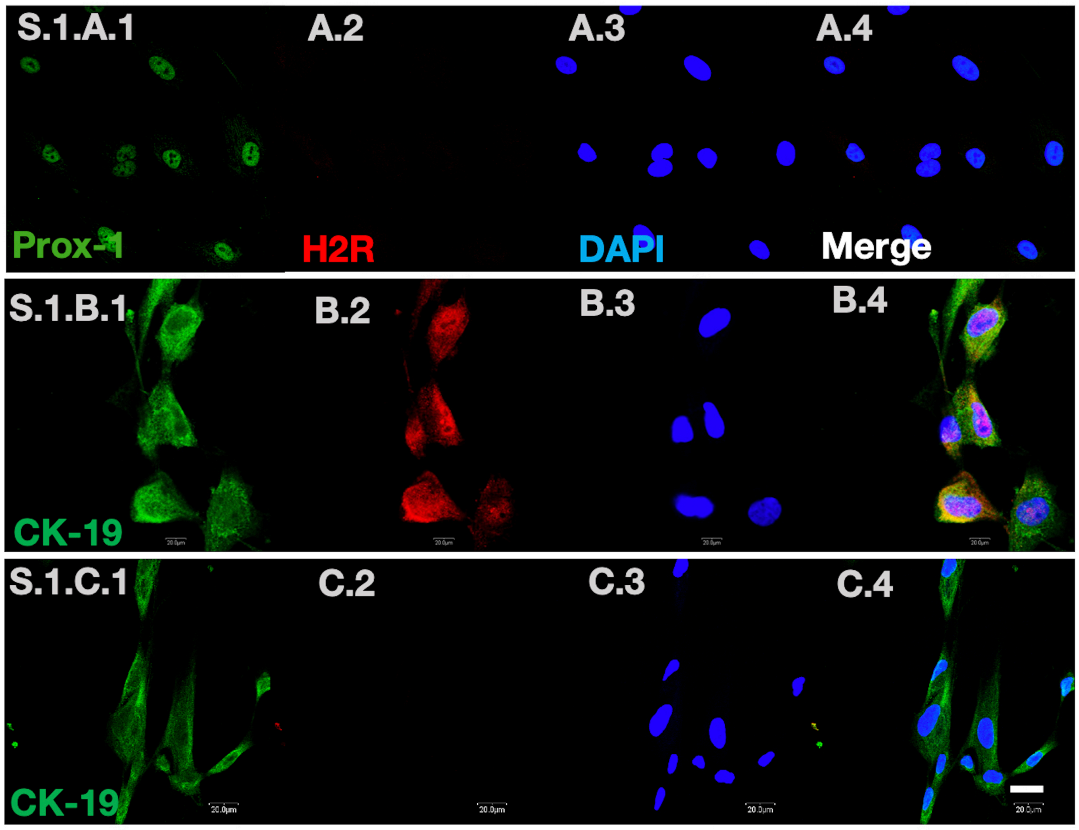

Fig. S1. A.1-4. Confocal images of LEC represents H2R negative control where immunofluorescence staining was conducted without H2R primary antibody. A.1. Prox-1 staining, A.2 represents absence of signal for H2R, A.3 DAPI and A.4 Merge. S.1.B.1-4: confocal images of HIBEC cell line, B.1 staining with CK19, B.2 staining with H2R B.3 DAPI staining and B.4 merge. S.C.1-4 represents negative control for H2R immunofluorescence staining in HIBEC. C.1: staining with CK19 C.2 absence of H2R signal C.3 DAPI C.4 Merge. Scale bar 20 μm

Biology Open • Supplementary information

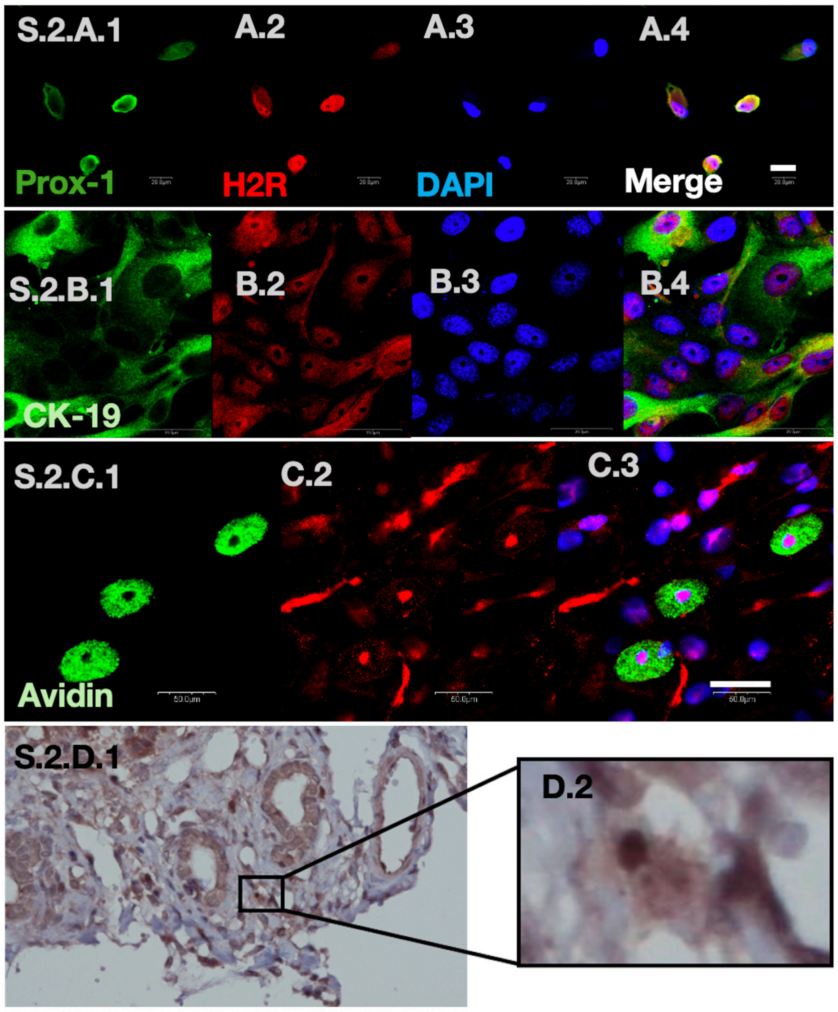

Fig. S2. A1-4. Confocal images of lymphatic fibroblast, S.A.1 Prox -1staining for lineage marker, A.2 H2R staining A.3 DAPI S.A.4. merge of all channels showing H2R nuclear localization S.2.B.1-4 Confocal images of rat cholangiocytes labelled with CK19 (green channel) H2R (red channel) Nucleus (DAPI) and B.4 overlay of merging all three channels. S.2.C.1-3 immunofluorescence staining of rat perilymphatic tissue for mast cell and H2R. C.1 is avidin 488 conjugate staining for perilymphatic mast cells. S.C.2 H2R staining in perilymphatic tissue S.C.3.Overaly of 488 and 647 channels, showing H2R positive signal in mast cells S.D.1 Immunohistochemical staining performed on the rat liver tissue. The tissue was stained with H2R antibody showing its nuclear localization, in the inset showing zoomed view, showing the localization pattern in S.D.2. All the representative images are of minimum three independent experiments. Scale bars 20 μm

Biology Open • Supplementary information

S3

BLAST analysis

RecName: Full=Beta-1 adrenergic receptor; AltName: Full=Beta-1 adrenoreceptor; Short=Beta-1 adrenoceptor [Homo sapiens]

Sequence ID: P08588.2 Length: 477 Number of Matches: 1

See 3 more title(s) See all Identical Proteins(IPG)

Range 1: 61 to 388 GenPept Graphics ▼ Next Match ▲ Previous Match

Score	Expect	Method	Identities	Positives	Gaps
186 bits(473)	3e-59	Compositional matrix adjust.	111/330(34%)	164/330(49%)	52/330(15%)

```
Query  21   ITVVLAVLILITVAGNVVVCLAVGLNRRLRNLTNCFIVSLAITDLLLGLLVLPFSAIYQL  80
            + +++A+++L+ VAGNV+V +A+    RL+ LTN FI+SLA  DL++GLLV+PF A   +
Sbjct  61   MGLLMALIVLLIVAGNVLVIVAIAKTPRLQTLTNLFIMSLASADLVMGLLVVPFGATIVV  120

Query  81   SCKWSFGKVFCNIYTSLDVMLCTASILNLFMISLDRYCAVMDPLRYPVLVTPVRVAISLV  140
              +W +G  FC ++TS+DV+  TASI  L +I+LDRY A+  P RY  L+T  R    +
Sbjct  121  WGRWEYGSFFCELWTSVDVLCVTASIETLCVIALDRYLAITSPFRYQSLLTRARARGLVC  180

Query  141  LIWVISITLSFLSIHLGW--NSRNETSKGNHTTSKCKVQVNEVYGLVDGLVTFYLPLLIM  198
             +W IS  +SFL I + W      +E  +  +    C     N  Y +    +V+FY+PL IM
Sbjct  181  TVWAISALVSFLPILMHWWRAESDEARRCYNDPKCCDFVTNRAYAIASSVVSFYVPLCIM  240

Query  199  CITYYRIFKVARDQAK--------------------------------------------  214
               Y R+F+ A+ Q K
Sbjct  241  AFVYLRVFREAQKQVKKIDSCERRFLGGPARPPSPSPSPVPAPAPPPGPPRPAAAAATAP  300

Query  215  ----RINHISSWKAATIREHKATVTLAAVMGAFIICWFPYFTAFVYRGLRGDDAINEVLE  270
                R       +   +RE KA  TL   +MG F +CW P+F A V +       + + + L
Sbjct  301  LANGRAGKRRPSRLVALREQKALKTLGIIMGVFTLCWLPFFLANVVKAFH-RELVPDRLF  359

Query  271  AIVLWLGYANSALNPILYAALNRDFRTGYQ  300
                WLGYANSA NPI+Y    + DFR  +Q
Sbjct  360  VFFNWLGYANSAFNPIIYCR-SPDFRKAFQ  388
```

Related Information

Gene - associated gene details

Identical Proteins - Identical proteins to P08588.2

Fig. S3. Result of BLAST analysis between H2R and Beta 1 adrenergic receptor

S.4.A BLAST analysis between H2R and other nuclear localized GPCRs

H2R vs Oxytocin receptor
Query cover: 75%
Evalue: 2e^-10
Percent Identical: 23.5%
Similarity:40%

H2R vs Beta 1 adrenergic receptor
Query cover: 77%
Evalue:3e^-59
Percent Identical: 34%
Similarity: 49%

H2R vs Prostaglandin E2 receptor
Query cover: 33%
Evalue: e^-8
Percent Identical: 26%
Similarity:48%

H2R vs Chemokine receptor 2
Query cover: 82%
Evalue:2e^-13
Percent Identical: 25%
Similarity: 45%

H2R vs Thyroid receptor alpha
No significant similarity

H2R vs Estrogen receptor alpha
No significant similarity

H2R vs H2R Isoform 1
Query cover: 100%
Evalue:0
Percent Identity: 100%

S.4.B

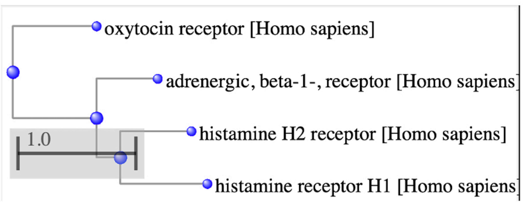

Fig. S4. A Summary of BLAST analysis comparison of nuclear localized GPCRs with H2R, with a positive control using H2R isoform 1. S.B Phylogenetic tree analysis between H2R, Beta 1 adrenergic receptor, oxytocin receptor along with H1R

Biology Open • Supplementary information
